# Joint QTL Mapping and Transcriptome Sequencing Analysis Reveal Candidate Genes for Salinity Tolerance in *Oryza sativa* L. ssp. Japonica Seedlings

**DOI:** 10.3390/ijms242417591

**Published:** 2023-12-18

**Authors:** Shuangshuang Li, Shanbin Xu, Jie Zheng, Haoqiang Du, Chong Li, Shen Shen, Shaoming Liang, Jingguo Wang, Hualong Liu, Luomiao Yang, Wei Xin, Yan Jia, Detang Zou, Hongliang Zheng

**Affiliations:** Key Laboratory of Germplasm Enhancement and Physiology & Ecology of Food Crop in Cold Region, Ministry of Education/College of Agriculture, Northeast Agricultural University, Harbin 150030, China; shuangshuangli@neau.edu.cn (S.L.); 18713599521@126.com (S.X.); zjyzdynb@126.com (J.Z.); 17390928573@163.com (H.D.); wangyi243081@163.com (C.L.); m15581871670@163.com (S.S.); leung0415@163.com (S.L.); 55190292@163.com (J.W.); liuhualongneau@163.com (H.L.); yaochang616@163.com (L.Y.); xinweineau@163.com (W.X.); jiayan_cool@126.com (Y.J.); zoudtneau@126.com (D.Z.)

**Keywords:** rice, QTL, transcriptome sequencing, salinity tolerance, candidate gene

## Abstract

Salinity stress is one of the major abiotic stresses affecting crop growth and production. Rice is an important food crop in the world, but also a salt-sensitive crop, and the rice seedling stage is the most sensitive to salt stress, which directly affects the final yield formation. In this study, two RIL populations derived from the crosses of CD (salt-sensitive)/WD (salt-tolerant) and KY131 (salt-sensitive)/XBJZ (salt-tolerant) were used as experimental materials, and the score of salinity toxicity (SST), the relative shoot length (RSL), the relative shoot fresh weight (RSFW), and the relative shoot dry weight (RSDW) were used for evaluating the degree of tolerance under salt stress in different lines. The genetic linkage map containing 978 and 527 bin markers were constructed in two RIL populations. A total of 14 QTLs were detected on chromosomes 1, 2, 3, 4, 7, 9, 10, 11, and 12. Among them, *qSST12-1*, *qSST12-2*, and *qRSL12* were co-localized in a 140-kb overlap interval on chromosome 12, which containing 16 candidate genes. Furthermore, transcriptome sequencing and qRT-PCR were analyzed in CD and WD under normal and 120 mM NaCl stress. *LOC_Os12g29330*, *LOC_Os12g29350*, *LOC_Os12g29390*, and *LOC_Os12g29400* were significantly induced by salt stress in both CD and WD. Sequence analysis showed that *LOC_Os12g29400* in the salt-sensitive parents CD and KY131 was consistent with the reference sequence (Nipponbare), whereas the salt-tolerant parents WD and XBJZ differed significantly from the reference sequence both in the promoter and exon regions. The salt-tolerant phenotype was identified by using two T_3_ homozygous mutant plants of *LOC_Os12g29400*; the results showed that the score of salinity toxicity (SST) of the mutant plants (*CR-3* and *CR-5*) was significantly lower than that of the wild type, and the seedling survival rate (SSR) was significantly higher than that of the wild type, which indicated that *LOC_Os12g29400* could negatively regulate the salinity tolerance of rice at the seedling stage. The results lay a foundation for the analysis of the molecular mechanism of rice salinity tolerance and the cultivation of new rice varieties.

## 1. Introduction

As the world population continues to grow, rice production will need to increase. Various abiotic conditions, including drought, salinity, heavy metals, and high temperature [1], affect rice growth and development, and salt stress is one of the most severe abiotic stresses. The effects of salt stress on rice mainly include osmotic stress and ionic toxicity, and ultimately lead to growth inhibition and yield loss [2]. Soil salinization is a global problem, mainly caused by irrational irrigation, climate change, and drought, and has affected more than 20% of arable land, and this percentage is expected to increase [3,4]. Therefore, improving the salinity tolerance of crops can promote the effective utilization of saline land and guarantee the sustainable development of agriculture. Growing rice in saline soils has been proven to be one of the effective ways to manage, improve, and utilize salinized soils [5,6]. However, rice is a salt-sensitive crop, and high salt stress can seriously affect the morphology of rice, including root damage, leaf curling, yellowing, reduction in the number of tillers per plant, reduction in biomass, shortening of plant height, decrease in thousand-grain weight, reduction in the number of spikelets per spike, and increase in the number of sterile florets, which ultimately leads to a decline in harvest index and yield [7]. Salt stress affects the physiological and biochemical processes of rice at all developmental stages from germination to senescence. Among them, the rice seedling stage is the most sensitive to salt stress, which directly affects the final yield formation [8]. Therefore, mining rice seedling salinity tolerance QTLs/genes is vital for breeding salt-tolerant rice varieties.

Salinity tolerance in rice is controlled by multiple genes, and scientists have cloned a series of genes involved in salinity tolerance [9]. Through linkage analysis, researchers used mapping populations derived from crosses between salt-sensitive and salt-tolerant varieties to identify a large number of quantitative trait loci (QTLs) associated with salinity tolerance in rice. For example, Chen et al. [10] identified a total of 23 salt-tolerant QTLs in the RIL population obtained from a cross between IR29 (salt-sensitive) and Pokkali (salt-tolerant), which resulted in the identification of a total of 23 QTLs for salinity tolerance in rice. Jahan et al. [11] utilized RIL populations derived from 93-11 and PA64s as experimental materials for QTL localization of six traits related to salinity tolerance, including shoot length, root length, shoot fresh weight, shoot dry weight, root fresh weight, and root dry weight, and identified a total of 38 QTLs. Lin et al. [12] identified a total of 11 QTLs for salinity tolerance in rice seedlings, utilizing the RIL populations derived from Nona Bokra (salt-tolerant) and Koshihikari (salt-sensitive), and using the days of seedling survival, the concentration of Na^+^ and K^+^, and the total amount of Na^+^ and K^+^ in shoots and roots as indicators. Kim et al. [13] identified a total of eight QTLs associated with salinity tolerance at the seedling stage of rice, with phenotypic contributions ranging from 10.20% to 13.90%, using 117 BC_3_F_5_ lines as the material, and survival rate, seedling height, leaf area, and seedling fresh and dry weight reduction rate as indicators. Takehisa et al. [14] conducted QTL analysis with 98 backcross-inbred lines (BIL) using seedling height, aboveground dry weight, and tiller number as indexes, and identified a total of 31 QTLs for rice seedling salinity tolerance, with phenotypic contributions ranging from 12.00–41.00%. To date, with the development of molecular marker technology and the construction of high-density linkage maps, a large number of genes in response to salt stress have been identified on 12 chromosomes. For instance, *SKC1* [15], *Saltol* [16], and *DST* [17] are the major genes associated with seedling stage under salt stress. Meanwhile, transcriptome sequencing analysis was able to identify differentially expressed genes (DEGs) responding to salt stress. For example, Zhu et al. [18] used bioinformatic approaches to perform a meta-analysis of three transcriptome datasets from salinity and control conditions, and, from a total of 28,432 expressed genes, to identify 457 core DEGs constitutively responding to salt. Geng et al. [19] found 551 salt stress-specific DEGs by comparing the transcriptome profiles of the two parents, Jileng 1 (salt-sensitive) and Milyang 23 (salt-tolerant); among them, fifteen DEGs located in stable QTL intervals were considered promising candidate genes for salinity tolerance.

In this study, two RIL populations derived from the crosses of CD (salt-sensitive)/WD (salt-tolerant) and KY131 (salt-sensitive)/XBJZ (salt-tolerant) were used as experimental materials for salinity-tolerant QTL analysis, combining transcriptome sequencing, qRT-PCR, and sequence analysis to screen for candidate genes within the co-localized QTL intervals, and then the knockout mutant plants were used for the preliminary functional verification of salinity tolerance. Finally, *LOC_Os12g29400* was confirmed to be a candidate gene for regulating salinity tolerance in rice.

## 2. Results

### 2.1. Phenotypic Variation

**Figure 1:**
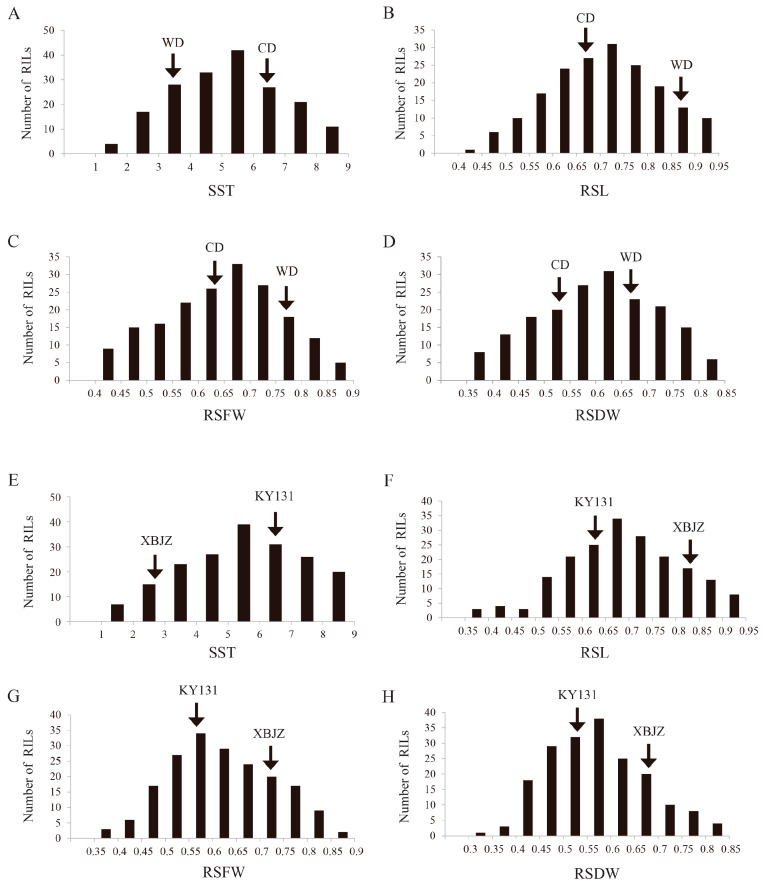
Phenotypic variation in the SST, RSL, RSFW, and RSDW in two RIL populations. (**A**–**D**) represent the SST, RSL, RSFW, and RSDW of RIL populations derived from CD/WD. (**E**–**H**) represent the SST, RSL, RSFW, and RSDW of RIL populations derived from KY131/XBJZ.

Statistical analysis of the data on four salinity tolerance phenotypes of SST, RSL, RSFW, and RSDW from two RIL populations showed that the range of variation of SST, RSL, RSFW, and RSDW in the RIL population derived from CD/WD crosses were 1.80–9.00, 0.44–0.95, 0.42–0.87, and 0.36–0.84, respectively. The coefficients of variation were 33.74%, 16.40%, 17.77%, and 19.36%, respectively (Table S1). The ranges of variation for SST, RSL, RSFW, and RSDW in the RIL population derived from the KY131/XBJZ were 1.20–8.80, 0.38–0.95, 0.37–0.87, and 0.35–0.85, with the coefficients of variation of 35.45%, 17.59%, 17.90%, and 18.09%, respectively (Table S1). All four salinity tolerance-related traits were significantly different among the two RIL populations’ respective parents, with the salt-tolerant parents WD and XBJZ showing a significantly lower SST than the salt-sensitive parents CD and KY131, and RSL, RSFW, and RSDW showing the opposite results (Table S1). Meanwhile, both RIL populations were contiguous in their distributions and showed significant superparental segregation, suggesting that all the traits exhibited quantitative trait inheritance characteristics (Figure 1). According to the heat map correlation analysis (Figure S1), the SST was negatively correlated with RSL, RSFW, and RSDW, and positively correlated with all RSL, RSFW, and RSDW.

### 2.2. Linkage Mapping for Salinity Tolerance

In the RIL population derived from the CD/WD cross, a total of seven QTLs associated with rice seedling salinity tolerance were detected on chromosomes 2, 4, 7, 10, 11, and 12, with LOD values ranging from 2.55 to 10.94 and the explained phenotypic variation ranging from 5.34% to 23.52%. Among them, there were two QTLs associated with SST, namely *qSST4* and *qSST12-1*; two QTLs associated with RSL, namely *qRSL2* and *qRSL10*; two QTLs associated with RSFW, namely *qRSFW4* and *qRSFW7*; and one QTL associated with RSDW, namely *qRSDW11* (Table 1). In the RIL population derived from the KY131/XBJZ, a total of seven QTLs associated with salinity tolerance at the seedling stage of rice were detected on chromosomes 1, 3, 4, 9, 11, and 12, with LOD values ranging from 2.77 to 9.26 and the explained phenotypic variation ranging from 5.89% to 17.83%. Among them, there were three QTLs associated with SST, namely *qSST1*, *qSST11*, and *qSST12-2*; two QTLs associated with RSL, namely *qRSL4* and *qRSL12*; one QTL associated with RSFW, namely *qRSFW9*; and one QTL associated with RSDW, namely *qRSDW3* (Table 2).

**Figure 2:**
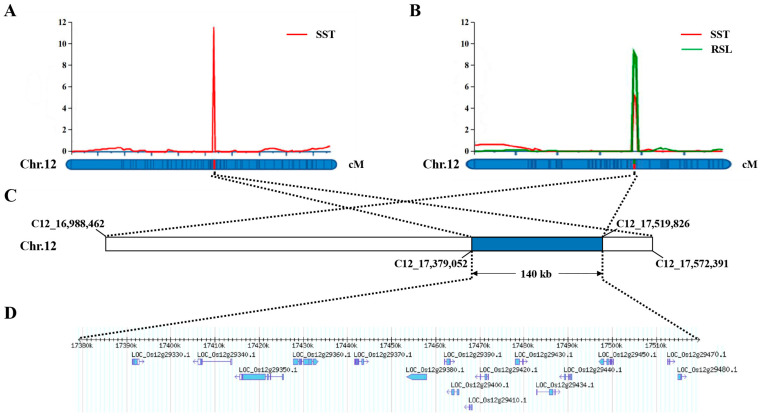
Identification of candidate genes by two RIL populations. (**A**) Salinity tolerance-related QTLs were detected in 189 RILs derived from CD/WD and mapped to the interval between markers C12_17379052 and C12_17572391 by linkage mapping. (**B**) Salinity tolerance-related QTLs were detected in 195 RILs derived from KY131/XBJZ and mapped to the interval between markers C12_16988462 and C12_17519826 by linkage mapping. (**C**) The two RIL populations co-located intervals. (**D**) The 140-kb region contained 16 genes.

Significantly, *qSST12-1*, *qSST12-2*, and *qRSL12* were co-located in the C12_17379052–C12_17519826 interval (140-kb overlap region) on rice chromosome 12 (Figure 2), and the *R*^2^ values were all above 10%. Therefore, we considered this interval as an essential salinity tolerance QTL in rice for further candidate gene mining.

### 2.3. RNA-Seq Statistics

**Figure 3:**
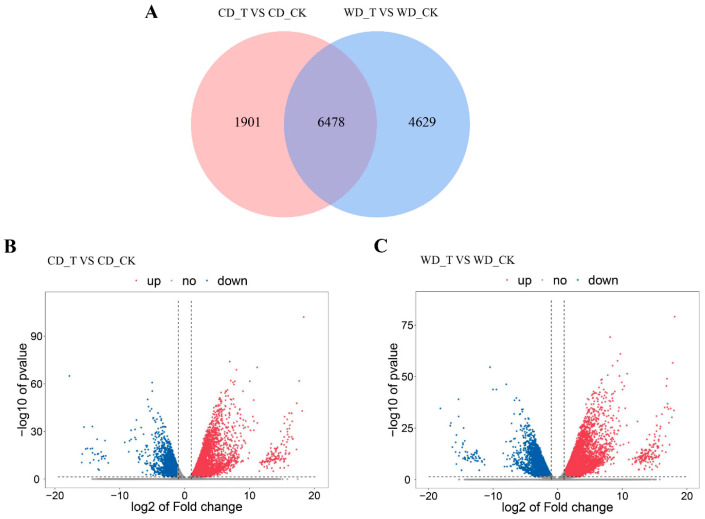
The differentially expressed genes (DEGs) in shoots. (**A**) Venn diagram of DEGs between two comparative groups. (**B**,**C**) Volcano plot of DEGs among different samples. X-axis and Y-axis present the value of log_2_^(ratio)^ and −log_10_^(FDR)^ of two comparative groups, respectively. Red (up-regulated) and blue (down-regulated) dots indicated that the genes have significant expression difference. CD_T: The samples of CD treated with 120 mM NaCl treatment for 12 h. CD_CK: The samples of CD treated with nutrient solution. WD_T: The samples of WD treated with 120 mM NaCl treatment for 12 h. WD_CK: The samples of WD treated with nutrient solution.

Differentially expressed genes (DEGs) of CD and WD under normal conditions and salt stress were analyzed by transcriptome sequencing. The results showed a total of 8379 DEGs in the CD_T VS CD_CK group, of which 5476 genes were up-regulated and 2903 genes were down-regulated for expression. There were 11,107 DEGs in the WD_T VS WD_CK group, of which 5184 genes were up-regulated and 5923 genes were down-regulated (Figure 3). Among them, seven DEGs (*LOC_Os12g29330*, *LOC_Os12g29340*, *LOC_Os12g29350*, *LOC_Os12g29390*, *LOC_Os12g29400*, *LOC_Os12g29410*, *LOC_Os12g29480*) in the CD_T VS CD_CK group and eight DEGs (*LOC_Os12g29330*, *LOC_Os12g29340*, *LOC_Os12g29350*, *LOC_Os12g29390*, *LOC_Os12g29400*, *LOC_Os12g29410*, *LOC_Os12g29430*, *LOC_Os12g29480*) in the WD_T VS WD_CK group were located at the 140-kb overlap region by QTL mapping.

**Figure 4:**
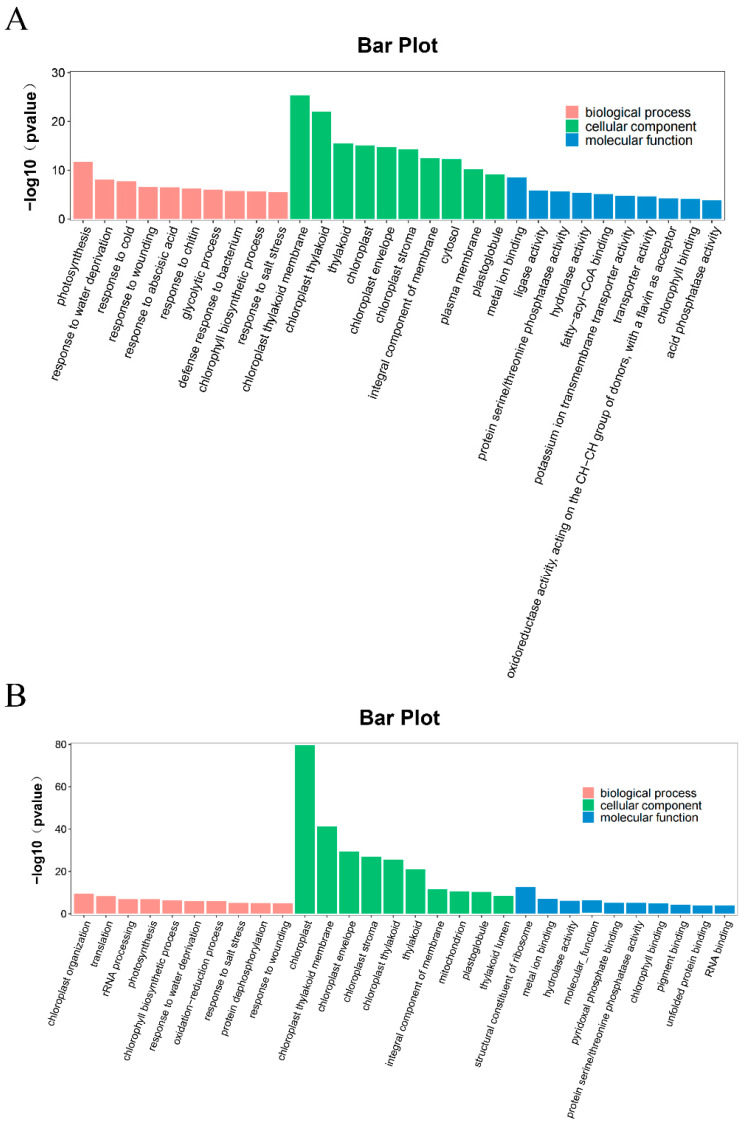
GO enrichment of differentially expressed genes in different comparison groups. (**A**) CD_T VS CD_CK. (**B**) WD_T VS WD_CK.

The differentially expressed genes in the two comparison groups, CD_T VS CD_CK and WD_T VS WD_CK, were analyzed for GO classification analysis (Figure 4). In the CD_T VS CD_CK group, the most significantly enriched biological process was GO:0015979 (photosynthesis); the most significantly enriched cellular component was GO:0009534 (chloroplast thylakoid membrane); and the most significantly enriched molecular function was GO:0046872 (metal ion binding) (Figure 4A). In the WD_T VS WD_CK group, the most significantly enriched biological process was GO:0009658 (chloroplast organization); the most significantly enriched cellular component was GO:0009507 (chloroplast); and the most significantly enriched molecular function was GO:0003735 (structural constituent of the ribosome) (Figure 4B).

**Figure 5:**
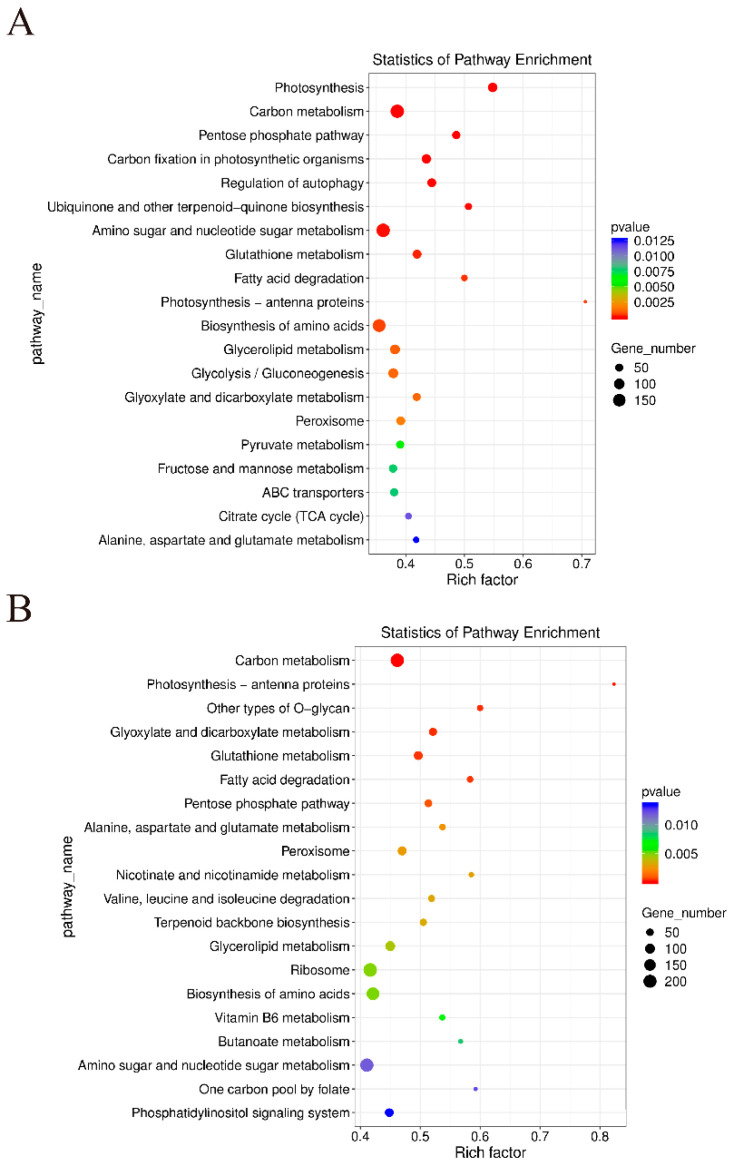
KEGG enrichment of differentially expressed genes in different comparison groups. (**A**) CD_T VS CD_CK. (**B**) WD_T VS WD_CK.

To further understand the biochemical metabolism and signaling pathways of the differentially expressed genes, KEGG enrichment analysis was performed on the differentially expressed genes in the two comparative groups, CD_T VS CD_CK and WD_T VS WD_CK (Figure 5). In the CD_T VS CD_CK group, ko00195 (photosynthesis), ko01200 (carbon metabolism), ko00030 (pentose phosphate pathway), ko00710 (carbon fixation in photosynthetic organisms), and ko04140 (regulation of autophagy) were the five KEGG pathways significantly enriched (Figure 5A). In the WD_T VS WD_CK group, ko01200 (carbon metabolism), ko00196 (photosynthesis-antenna proteins), ko00514 (other types of O-glycan), ko00630 (glyoxylate and dicarboxylate metabolism), and ko00480 (glutathione metabolism) were the five KEGG pathways significantly enriched (Figure 5B).

### 2.4. Candidate Gene Mining

**Figure 6:**
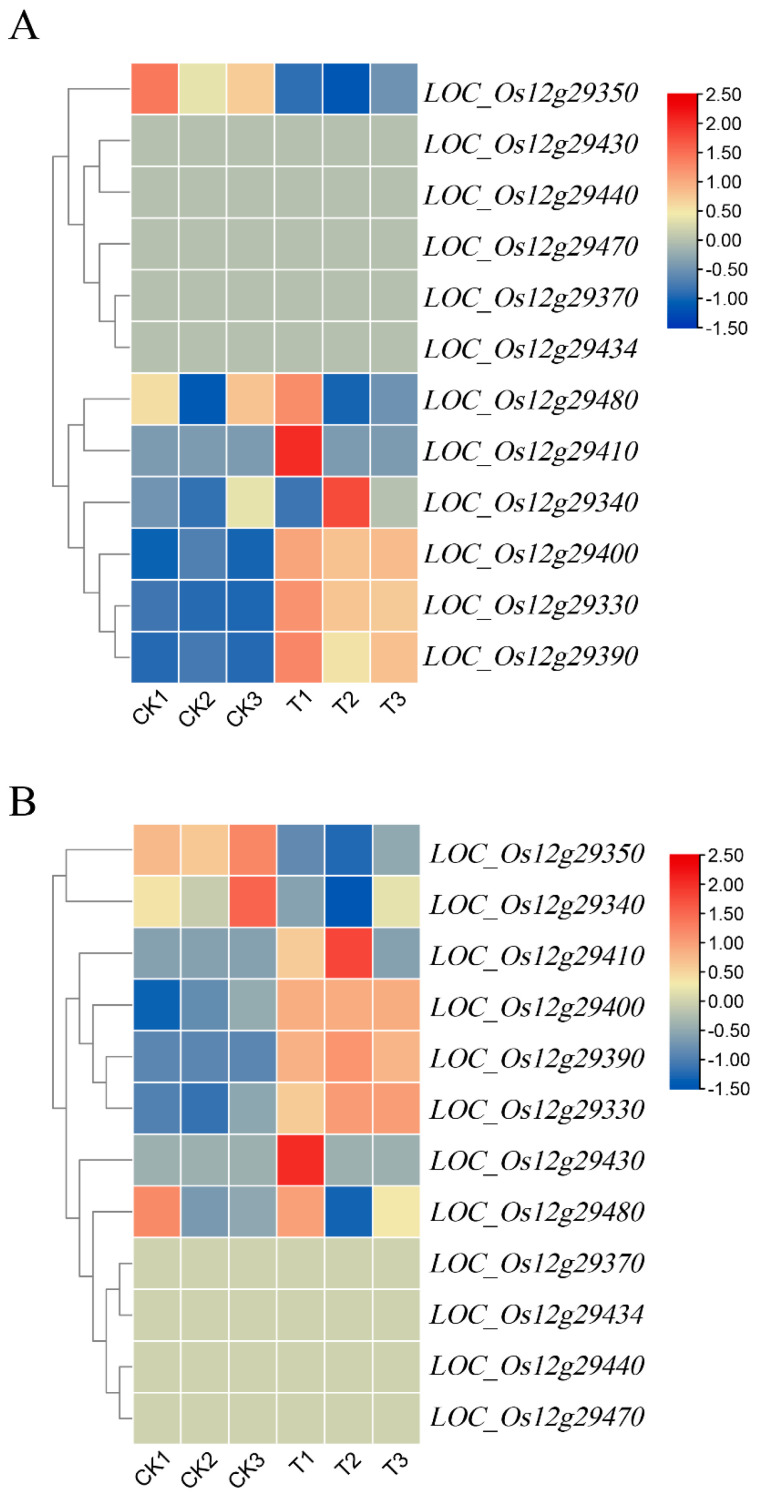
Heatmap of gene expression within candidate intervals. (**A**) The heatmap of expression of 12 genes in the interval with normal and salt treatment of CD; (**B**) The heatmap of expression of 12 genes in the interval with normal and salt treatment of WD; CK: control group (untreated); T: test group (salt-treated).

**Figure 7:**
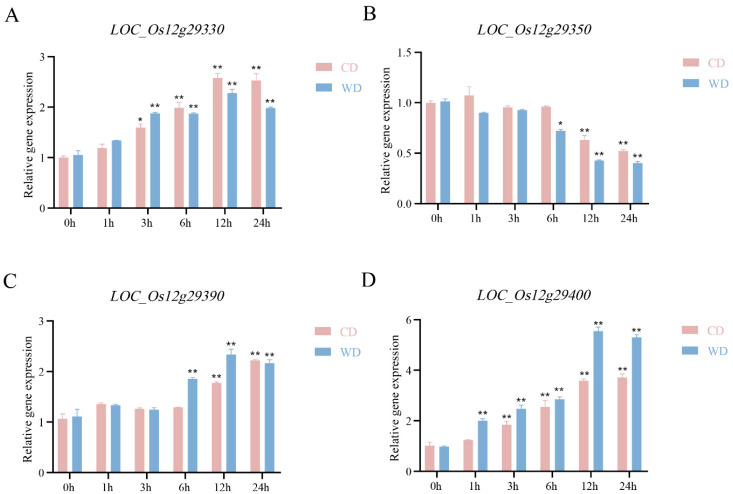
Expression patterns of the four genes under normal growth conditions and salinity stress. (**A**–**D**) represent the gene expression of *LOC_Os12g29330*, *LOC_Os12g29350*, *LOC_Os12g29390*, and *LOC_Os12g29400* under normal growth conditions and salinity stress. (* *p* < 0.05,** *p* < 0.01, Students’ *t*-test).

According to Rice Genome Annotation Project (RGAP), the 140-kb overlap region contains 16 candidate genes, including eight functionally annotated genes, three genes encoding proteins with unknown functions, one gene encoding hypothetical proteins, and four genes encoding retrotransposons (Figure 2D and Table S2). Excluding the four genes encoding retrotransposons, seven of the remaining 12 genes were induced to express in CD under salt treatments and eight genes were induced to express in WD under salt treatments by transcriptome sequencing analysis (Figure 6), and the expression of four genes, *LOC_Os12g29330*, *LOC_Os12g29350*, *LOC_Os12g29390*, and *LOC_Os12g29400*, in both CD and WD were significantly induced by salt stress. Therefore, four genes were considered as salinity tolerance candidate genes for further analysis. qRT-PCR analysis showed that the expression of these four genes was induced by salt stress in both CD and WD, and three genes (*LOC_Os12g29330*, *LOC_Os12g29390*, *LOC_Os12g29400*) were up-regulated for expression by salt stress, and *LOC_Os12g29350* was down-regulated for expression by salt stress, with the expression of *LOC_Os12g29400* being induced by the greatest degree of salt stress (Figure 7). Gene expression trends analyzed by qRT-PCR were consistent with those of the transcriptome sequencing results.

After observing these results, we sequenced these four genes (*LOC_Os12g29330*, *LOC_Os12g29350*, *LOC_Os12g29390*, *LOC_Os12g29400*) from four parents (CD, WD, KY131, and XBJZ). The results showed that the *LOC_Os12g29400* in CD (salt-sensitive) and KY131 (salt-sensitive) was consistent with the reference sequence (Nipponbare). In contrast, *LOC_Os12g29400* in WD (salt-tolerant) had 17 SNPs and two indel (1-bp deletion and 2-bp insertion) mutations in the promoter region, two indel (6-bp deletion and 3-bp deletion) mutations in the first exon, and one indel (2-bp deletion) mutation in the 3′UTR region. *LOC_Os12g29400* in XBJZ (salt-tolerant) was present with one SNP and a 1-bp insertion mutation in the promoter region, a 6-bp deletion and one SNP mutation in the first exon region, one SNP mutation in the second exon, and one SNP mutation in the 3′UTR (Figure S5). After comparison, it was found that *LOC_Os12g29400* in both salt-tolerant parents WD and XBJZ had a 6-bp deletion in the first exon region (Figure S4), which might be the functional site for its high salinity tolerance, to be further verified. No mutations were detected in the other three genes (*LOC_Os12g29330*, *LOC_Os12g29350*, *LOC_Os12g29390*) in four parents.

### 2.5. Validation of the LOC_Os12g29400 Mutant

**Figure 8:**
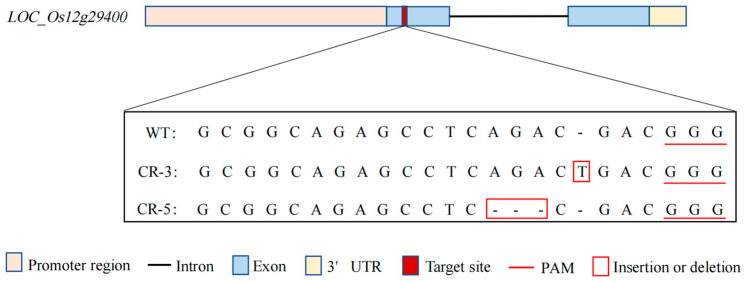
Target sequences of WT (Nipponbare) and knockout mutant plants (*CR-3*, *CR-5*).

**Figure 9:**
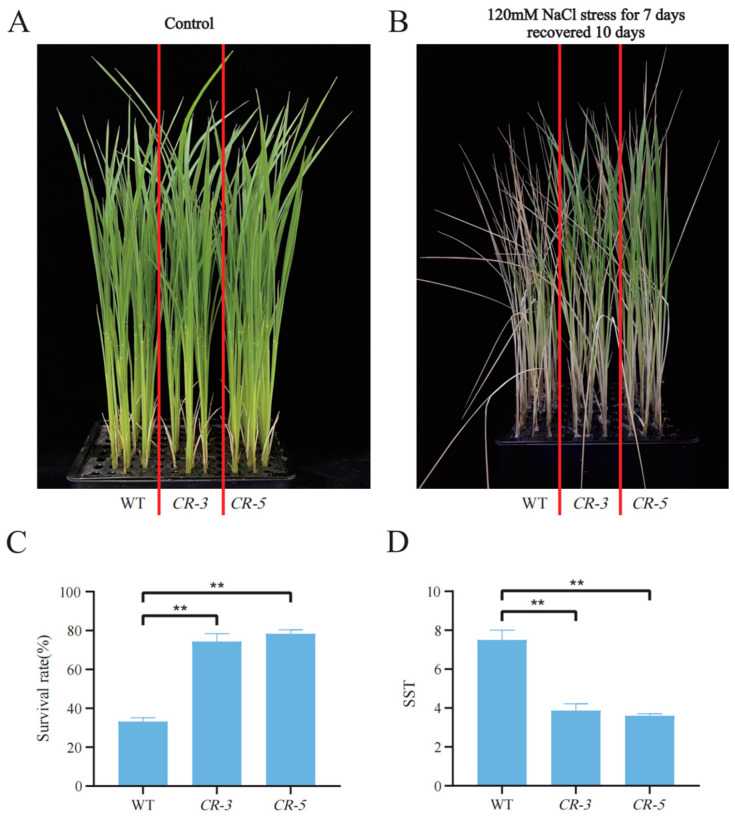
Salinity tolerance phenotype of WT (Nipponbare) and knockout mutant plants (*CR-3*, *CR-5*). (**A**) Phenotype of the control group; (**B**) Phenotypes of the salt-treated group; (**C**) Survival statistics of WT (Nipponbare), *CR-3,* and *CR-5* in the salt treatment group; (**D**) The SST of WT (Nipponbare), *CR-3*, and *CR-5* in the salt treatment group. The red line distinguishes wild-type material from transgenic material. (** *p* < 0.01; Students’ *t*-test).

To investigate the relationship between *LOC_Os12g29400* and salinity tolerance in rice, we screened two homozygous knockout mutant types of plants, *CR-3* with a one-base (T) insertion and *CR-5* with a three-base (AGA) deletion at the target site (Figure 8), and propagated them to the T_3_ generation for subsequent analysis. Wild-type (Nipponbare), *CR-3*, and *CR-5* grown to the two leaves and one heart stage were divided into two groups, one with normal nutrient solution conditions and the other with an additional 120 mM NaCl for salt stress treatment for 7 days and recovering for 10 days, to determine their salinity tolerance phenotype. The results showed that, under normal conditions, there was no significant difference in the growth of mutant and wild-type rice seedlings (Figure 9A), while under salt-treated conditions, the mutant plants *CR-3* and *CR-5* were more salt-tolerant, with an average SST of 3.9 and 3.6, and an average SSR of 74.3% and 78.3%, compared with an average SST of 7.5 and an average SSR of 33.2% in the wild type (Figure 9), indicating that the *LOC_Os12g29400* knockout enhanced salinity tolerance in rice.

## 3. Discussion

Two RIL populations were utilized to locate 14 QTLs, including *qSST1*, *qRSL2*, *qRSDW3*, *qRSL4*, *qRSFW4*, *qSST4*, *qRSFW7*, *qRSFW9*, *qRSL10*, *qSST11*, *qRSDW11*, *qSST12-1*, *qSST12-2*, and *qRSL12*. By comparing with the already reported rice salinity tolerance QTLs/genes, 10 QTLs were found to be located in the same interval as known rice salinity tolerance QTLs, and five QTLs contained known rice salinity tolerance genes within their intervals (Table 1 and Table 2). Three QTLs, *qRSFW4*, *qSST11*, and *qRSDW11,* might be new rice salinity tolerance QTLs. The QTL *qSST1* was located in the same interval with K^+^ concentration-related *qSKC1* [27], and included the known rice salinity tolerance gene *OsKAT1* [28], which could place the function as an endocytosis-type potassium ion channel. The QTL *qRSL2* was located in the same interval with Na^+^ concentration-related *qNA-2a* [22], and contained known rice salinity tolerance genes *OsMADS27* and *OsPMS1* [23,24], of which *OsMADS27* was able to regulate rice salinity tolerance positively and was associated with nitrate concentration, and *OsPMS1* was a mismatch repair protein present in rice, and inhibition of its expression would improve rice salinity tolerance. The QTL *qRSFW7* included the known rice salinity tolerance gene *ZFP245*. Huang et al. [26] found that the expression of *ZFP245* in tobacco improved the salinity tolerance of transgenic plants through transgenic assays. The QTL qRSFW9 contained *qGP-7-2* associated with germination rate under salt stress [29], while qRSFW9 also included the known rice salinity tolerance genes *OsHTAs* and *OsSIPP2C1* [30,31], the expression of both of which were induced by salt stress. The QTLs *qSST12-1*, *qSST12-2*, and *qRSL12* were all located in the same interval as the Na^+^/K^+^-related *qSNK-12* [21]. The above findings support our results.

Transcriptome analysis showed that the expression of seven and eight genes was induced by salt stress in CD and WD, respectively, and the expression of four genes was significantly induced by salt stress in both varieties. qRT-PCR analysis revealed that among the four genes, the expression of *LOC_Os12g29400* was the most induced by salt stress. In the previous study, using cDNA microarray and RNA get-blot analyses, Rabbani et al. [32] found that the expression of *LOC_Os12g29400* can be induced by high salt, low temperature, dehydration, and ABA at the rice seedling stage. We further found that *LOC_Os12g29400* of the salt-tolerant parents WD and XBJZ differed from the salt-sensitive parents CD and KY131 in the promoter and exon regions, and both had a 6-bp deletion of indel in the first exon by sequence analysis. The seedling salinity tolerance of knockout mutant plants of *LOC_Os12g29400* was significantly higher than that of the wild type. The above results preliminarily proved that *LOC_Os12g29400* can negatively regulate salinity tolerance in rice, and its clear functions of salinity tolerance and molecular mechanism resolution need to be further investigated.

We found that *LOC_Os12g29400* contains a GRAM structural domain. The GRAM structural domain is highly conserved and is present in glucosyltransferases, myotubularins, and other putative membrane-associated proteins [33], and that genes containing GRAM structural domains in plants are involved in the perception and regulation of environmental and hormonal signals under various stress conditions [34,35,36,37]. Tiwari et al. [38] found that the expression of partial GRAM genes in rice could be induced by salt stress, drought stress, and ABA. This suggests that GRAM structural domain genes in rice may play an important role in salt stress tolerance, while the relatively few studies on GRAM structural domain genes in rice have not been deeply refined. Therefore, further studies are needed to investigate the role of GRAM structural domain genes in abiotic stress tolerance, especially salt stress tolerance in the future.

## 4. Conclusions

A total of 14 QTLs were detected by using two RIL populations. Among them, *qSST12-1*, *qSST12-2*, and *qRSL12* were co-located in the interval C12_17379052–C12_17519826 on chromosome 12, and the combination of transcriptome sequencing and qRT-PCR revealed that *LOC_Os12g29400* expression was induced to the highest degree by salt stress. Sequence analysis showed that *LOC_Os12g29400* in the salt-sensitive parents CD and KY131 was consistent with the reference sequence (Nipponbare), while *LOC_Os12g29400* in the salt-tolerant parents WD and XBJZ differed significantly from the reference sequence both in the promoter and exon regions. Rice seedling experiments showed that the salinity tolerance of *LOC_Os12g29400* homozygous mutant plants was significantly stronger than that of the wild type, which indicated that *LOC_Os12g29400* could negatively regulate the salinity tolerance of rice.

## 5. Materials and Methods

### 5.1. Plant Materials

Two RIL populations were used as experiment materials, a RIL population containing 189 lines derived from a cross between the salinity-sensitive variety CD and the salinity-tolerant variety WD, and a RIL population containing 195 lines derived from a cross between the salinity-sensitive variety Kongyu131 and the salinity-tolerant variety Xiaobaijingzi [39]. The salinity tolerance phenotypes of CD, WD, KY131, and XBJZ are shown in Figure S2. All RILs were planted at the Experimental Station of Northeast Agricultural University, Heilongjiang Province, China (45°50′ N, 126°40′ E).

### 5.2. Salinity Tolerance Evaluation at the Seedling Stage

All materials were placed in an oven at 55 °C for 48 h to break seed dormancy. The full rice seeds were selected and disinfected with 3% NaClO solution for 20 min, after which the seeds were washed three times with sterile water to remove the disinfection solution residue from the seed surface. The seeds were placed in a constant temperature incubator at 30 °C and hydroponically cultivated for 4 days, and then the experiment was divided into two groups, named T_1_ and T_2_ groups, both set up with three replications.

In group T_1_, 64 uniformly germinated rice seeds of each variety were equally divided into treatment and control groups, sown in 96-well plates with one seed in each well, and grown hydroponically with Yoshida nutrient solution. The germinated seeds were transferred to an artificial climate chamber and incubated at 25 and 23 °C, and 14 h of light and 10 h of dark cycle, respectively. When the seedlings grew to two leaves and one heart, the control group was treated with normal application of nutrient solution and the treatment group was treated with salt stress, pretreated with sodium chloride (NaCl) at a concentration of 50 mmol/L for 3 days, then the seedlings were treated with NaCl at a concentration of 120 mmol/L for 7 days, and five plants of each line in the two RIL populations and parental lines were randomly selected for the determination of the shoot length (SL), the shoot fresh weight (SFW), and the shoot dry weight (SDW) under the control conditions and salt treatment. The relative shoot length (RSL), the relative shoot fresh weight (RSFW), and the relative shoot dry weight (RSDW) were further calculated as follows: Relative value = (phenotype value under salt treatment)/(phenotype value under control condition). Meanwhile, in group T_2_, 100 uniformly germinated seeds of each variety were grown with Yoshida nutrient solution, with the same salt treatment as the T_1_ group. The culture medium was replaced every day, and replaced with the same medium as the control group after 7 days. After 10 days of recovery growth, the salt damage levels of 10 seedlings in the salt treatment group were randomly determined, and the scores of salinity toxicity (SST) were classified as 1, 3, 5, 7, and 9, with reference to the standards of the International Rice Research Institute.

### 5.3. QTL Mapping for Salinity Tolerance

In this study, the two RIL populations were genotyped by 10 K Array targeted sequencing technology from MOLBREEDING Biotechnology Company (Shijiazhuang, China). Multiple SNP markers with the same genotype were combined into a “BIN” after biparental polymorphism analysis and de-redundancy analysis by the IciMapping-Bin project. A genetic linkage map containing 978 bin markers was constructed using 189 RILs derived from CD and WD, which covered 2465.33 cM of the rice genome with an average distance of 2.52 cM between markers (Figure S3). Another genetic linkage map containing 527 bin markers was constructed using 195 RILs derived from KY131 and XBJZ, which covered 1874.85 cM of the rice genome with an average distance of 3.56 cM between markers (Figure S4). The Genotype data were obtained from 10 K Array genotyping by target sequencing (GBTS) by MOLBREEDING Biotechnology Company (Shijiazhuang, China). QTL mapping was performed using the inclusive composite interval mapping method (ICIM) implemented in ICIMapping (Ver.4.2), and the threshold value was set to LOD > 2.5, according to a pre-laboratory study [40].

### 5.4. RNA-Seq

The parents CD (salinity-sensitive) and WD (salinity-tolerant) were selected for transcriptome sequencing. Firstly, when the seeds grew to two leaves and one heart, they were divided into two groups. One group was given normal application of nutrient solution, and the other group was treated with 120 mmol/L NaCl with salt treatment for 12 h. The aboveground tissue of rice in the two groups was sampled separately; the experiment was set up with three replications. RNA was extracted using TranZol Up RNA kit (TransGen Biotech, Beijing, China). The amount and purity of total RNA were then quality controlled using NanoDrop ND-1000. The fragmented RNA was synthesized into cDNA using reverse transcriptase using HiFiScript cDNA Synthesis Kit (CWBio, Beijing, China). qRT-PCR was performed using 2 × Fast qPCR Master Mix (DINING, Beijing, China) and sequencing was performed using Illumina Novaseq™6000. The obtained CleanData was compared to the genome using HISAT2 [41]. Genes and transcripts were assembled using StringTie, and the assembly results of all the samples were combined, and the final assembly annotation results were obtained using gff compare to detect the comparison of transcripts with reference annotations. The ballgown package provided file input for FPKM quantification [42,43]. Significant differences between samples were analyzed using the R package edgeR [44].

### 5.5. Identification of Candidate Genes by Gene Expression and Sequence Analysis

The expression levels of the four genes of CD and WD were verified by qRT-PCR analysis under salinated and normal conditions. qRT-PCR analysis was performed using Roche LightCycler96. All primer sequences are shown in Table S3. The CDS and promoter regions of CD and WD candidate genes were cloned using PCR. Sequence comparisons were performed using DNAMAN.

### 5.6. LOC_Os12g29400 Mutant Plants

Seeds of the *LOC_Os12g29400* knockout mutant of the T1 generation in the background of Nipponbare were obtained from BIOGLE GENETECH company (http://www.biogle.cn/, accessed on 9 November 2023), while the mutant seeds were obtained using the CRISPR/Cas9 method. Two T1 generation homozygous mutant plants were screened by PCR amplification of sequences at the target site and propagated to T3 by selfing for the seedling salinity tolerance experiment. The wild type (Nipponbare) and the T3 generation homozygous mutant plants were used as experimental materials, divided into two groups, and when they grew up to two leaves and one heart, nutrient solution was applied normally to one group, and 120 mmol/L NaCl was applied to the other, and the treatment was carried out for 7 days, and the recovery was carried out for 10 days, to determine the score of salinity toxicity (SST) and the seedling survival rate (SSR), and the experiment was set up with three replications.

## Figures and Tables

**Table 1:** QTLs for SST, RSL, RSFW, and RSDW detected by linkage mapping analysis in 189 RILs derived from CD/WD.

Traits	QTLs	Left Marker	Right Marker	Chr.	LOD	*R*^2^ (%)	Additive Effect	Known QTLs	Known Genes
SST	*qSST4*	C4_32625360	C4_32680426	4	2.98	5.85	−0.41	*qDWT4.32* [20]	
	*qSST12-1*	C12_17379052	C12_17572391	12	10.94	23.52	−0.83	*qSNK-12* [21]	
RSL	*qRSL2*	C2_21864234	C2_24239570	2	4.57	9.70	0.04	*qNA-2a* [22]	*OsMADS27* [23] *OsPMS1* [24]
	*qRSL10*	C10_2404205	C10_2549079	10	2.55	5.34	−0.03	*qPF10.2* [25]	
RSFW	*qRSFW4*	C4_26190239	C4_26579339	4	2.66	5.90	−0.03		
	*qRSFW7*	C7_23130700	C7_24094627	7	4.30	10.47	−0.04		*ZFP245* [26]
RSDW	*qRSDW11*	C11_23355381	C11_23543062	11	4.70	11.67	−0.04		

*R*^2^ (%): Phenotypic variance explained.

**Table 2:** QTLs for SST, RSL, RSFW, and RSDW detected by linkage mapping analysis in 195 RILs derived from KY131/XBJZ.

Traits	QTLs	Left Marker	Right Marker	Chr.	LOD	*R*^2^ (%)	Additive Effect	Known QTLs	Known Genes
SST	*qSST1*	C1_31761401	C1_32601806	1	2.96	5.89	−0.48	*qSKC1* [27]	*OsKAT1* [28]
	*qSST11*	C11_25364222	C11_25595354	11	3.88	7.68	0.55		
	*qSST12-2*	C12_16988462	C12_17519826	12	5.25	10.60	−0.04	*qSNK-12* [21]	
RSL	*qRSL4*	C4_21835179	C4_22375729	4	3.71	7.19	0.03	*qDTF4.1s* [27]	
	*qRSL12*	C12_16988462	C12_17519826	12	9.26	17.83	−0.06	*qSNK-12* [21]	
RSFW	*qRSFW9*	C9_9364678	C9_9790798	9	2.77	7.40	−0.03	*qGP-7-2* [29]	*OsHTAs* [30] *OsSIPP2C1* [31]
RSDW	*qRSDW3*	C3_26113568	C3_28667135	3	2.77	6.35	−0.03	*qCHL3.26* [20]	*OsBIHD1* [20]

*R*^2^ (%): Phenotypic variance explained.

## Data Availability

Data are contained within the article.

## Supplementary Materials

The supporting information can be downloaded at: https://www.mdpi.com/article/10.3390/ijms242417591/s1.

## Abbreviations

RIL: recombinant inbred lines; SST: score of salinity toxicity; RSL: relative shoot length; RSFW: relative shoot fresh weight; RSDW: relative shoot dry weight; SSR: seedling survival rate; GO: Gene ontology; KEGG: Kyoto Encyclopedia of Genes and Genomes.
