# Supplementary figures and images for: Joint QTL Mapping and Transcriptome Sequencing Analysis Reveal Candidate Genes for Salinity Tolerance in Oryza sativa L. ssp. Japonica Seedlings
Source: Int J Mol Sci. 2023 Dec 18;24(24):17591. doi: 10.3390/ijms242417591 (PMC10743832; doi:10.3390/ijms242417591)

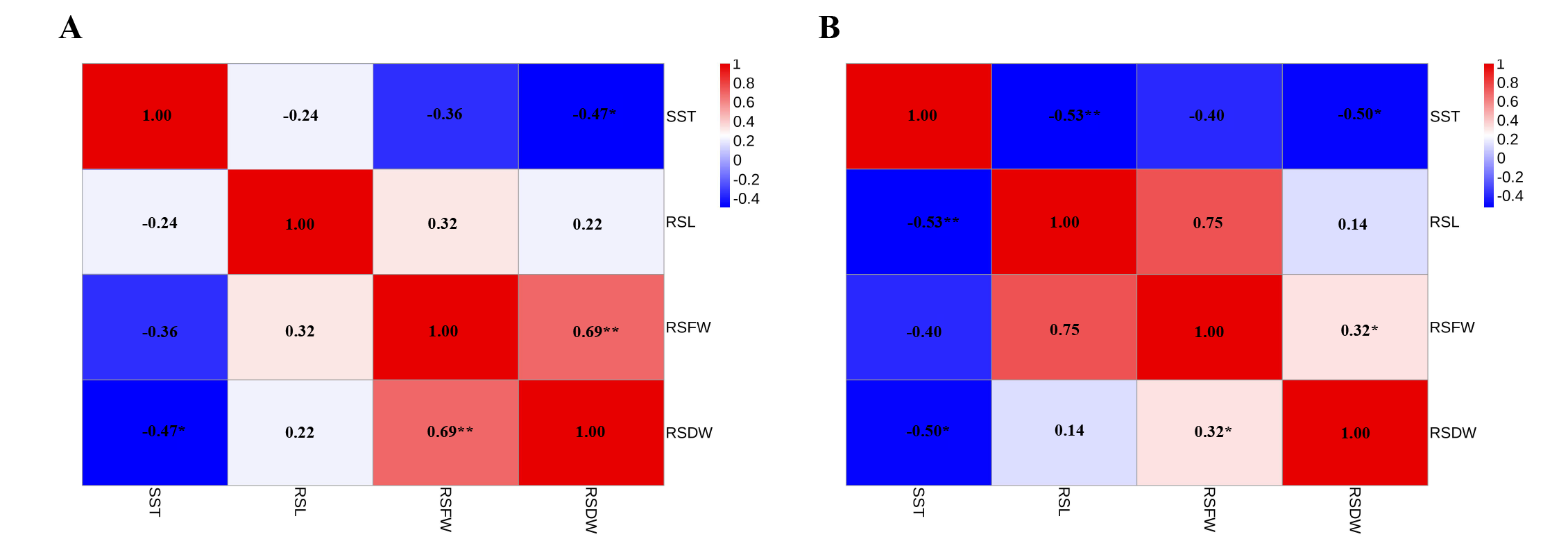

Supplement: Supplementary file 1 [file ijms-24-17591-s001.zip › Supplementary File(s)/Figure S1.tif]

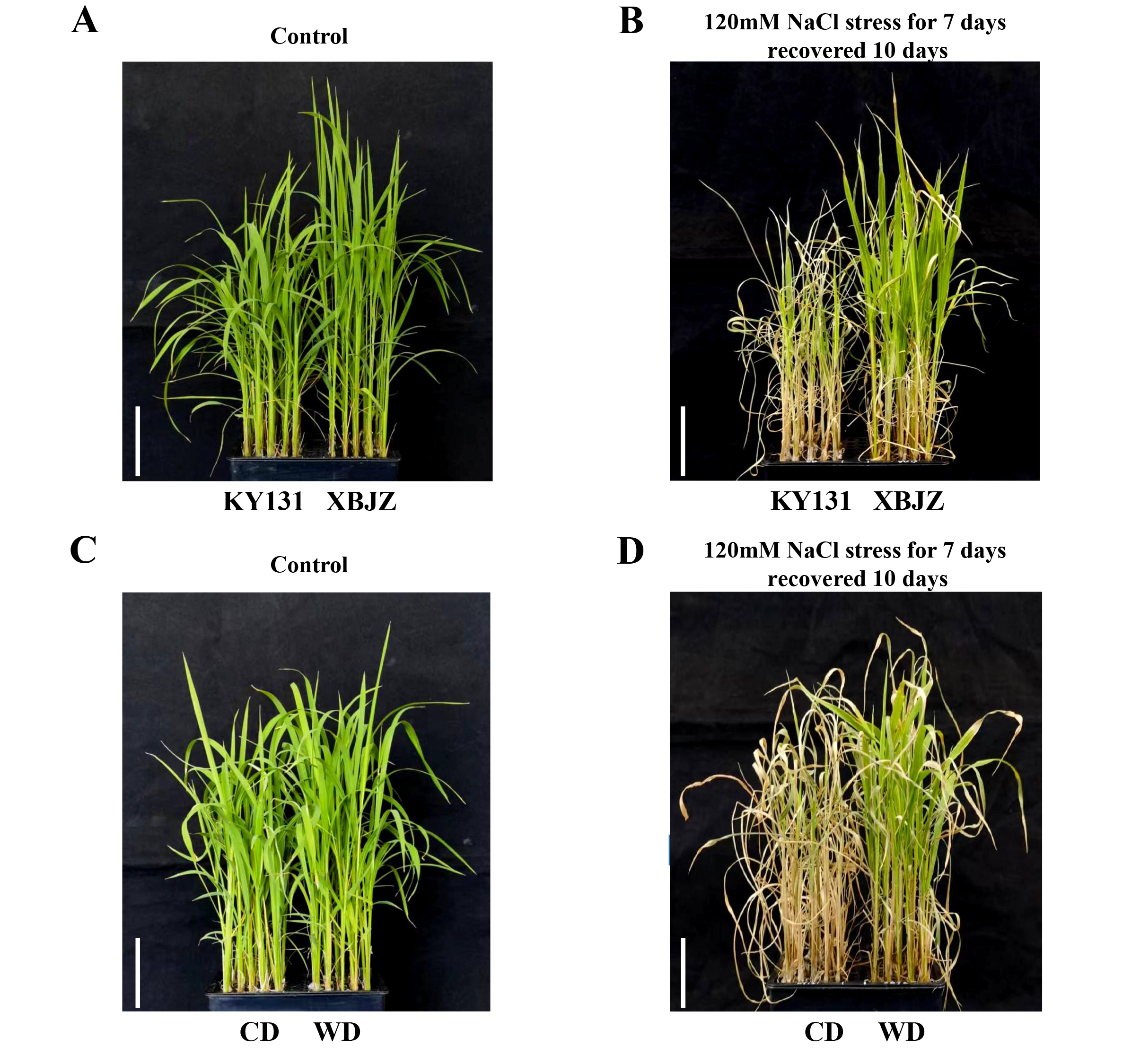

Supplement: Supplementary file 1 [file ijms-24-17591-s001.zip › Supplementary File(s)/Figure S2.tif]

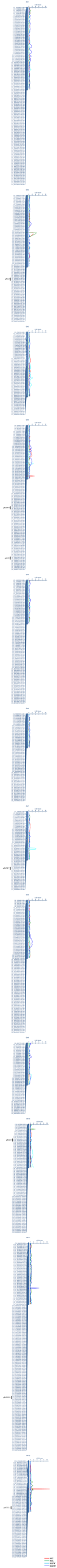

Supplement: Supplementary file 1 [file ijms-24-17591-s001.zip › Supplementary File(s)/Figure S3.tif]

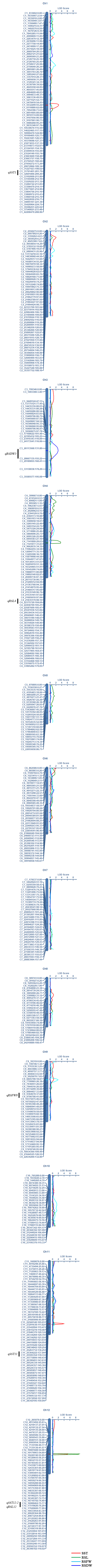

Supplement: Supplementary file 1 [file ijms-24-17591-s001.zip › Supplementary File(s)/Figure S4.tif]

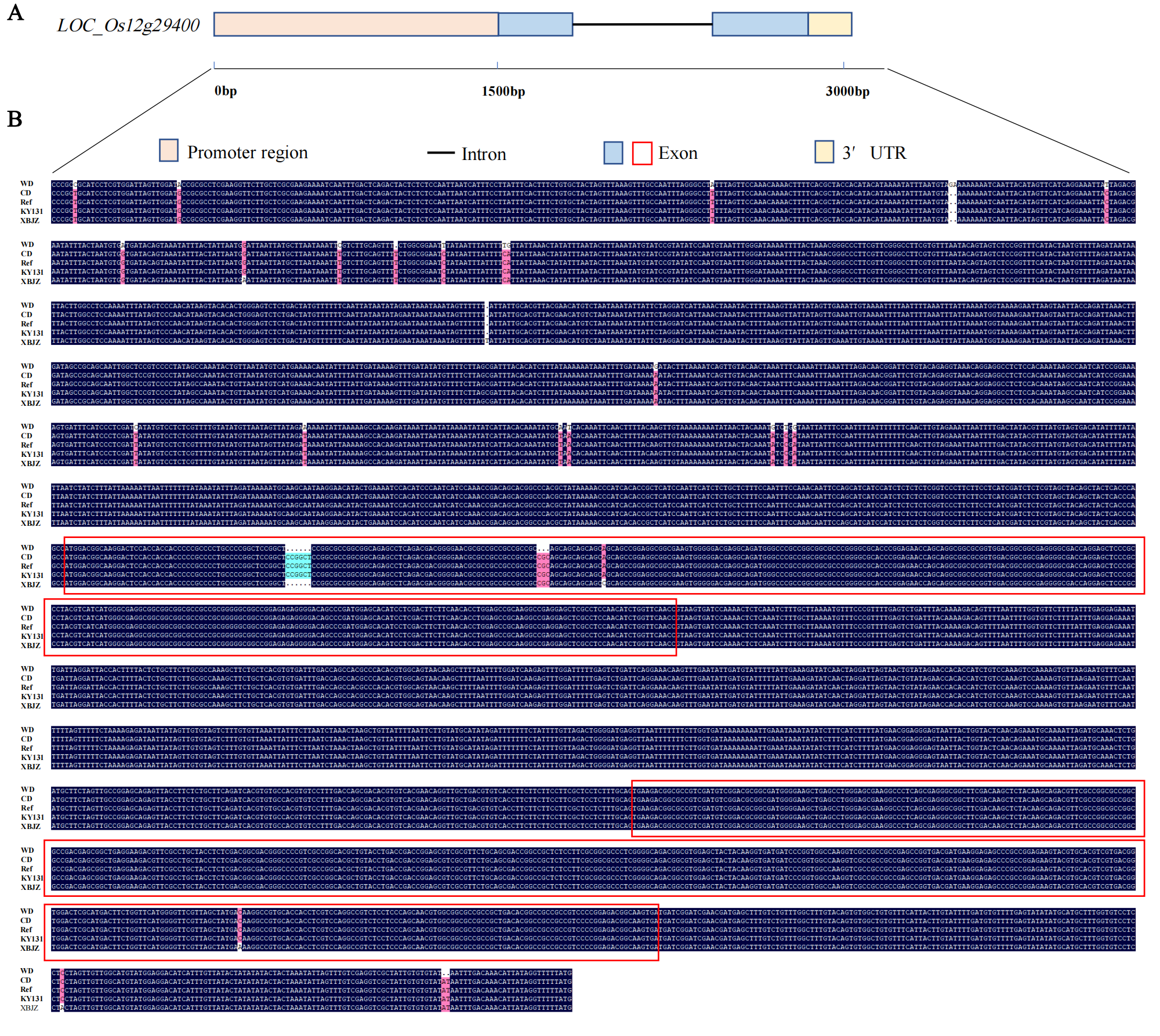

Supplement: Supplementary file 1 [file ijms-24-17591-s001.zip › Supplementary File(s)/Figure S5.tif]
